# Integrative analysis indicates the potential values of ANKRD53 in stomach adenocarcinoma

**DOI:** 10.1007/s12672-024-01054-5

**Published:** 2024-05-27

**Authors:** Chunjing Jin, Xu Lu, Minfeng Yang, Shiqiang Hou

**Affiliations:** 1 Laboratory Medicine Center, The Affiliated Chuzhou Hospital of Anhui Medical University, The First People’s Hospital of Chuzhou, Chuzhou, China; 2 Department of General Surgery, The Affiliated Chuzhou Hospital of Anhui Medical University, The First People’s Hospital of Chuzhou, Chuzhou, China; 3 School of Public Health, Nantong University, Nantong, China; 4 Department of Health Technology and Informatics, The Hong Kong Polytechnic University, Kowloon, Hong Kong SAR China; 5 Department of Neurosurgery, The Affiliated Chuzhou Hospital of Anhui Medical University, The First People’s Hospital of Chuzhou, Chuzhou, China

**Keywords:** ANKRD53, Stomach adenocarcinoma, Biomarker, Prognosis, Immunity

## Abstract

**Background:**

Ankyrin repeat domain 53 (ANKRD53) plays an important role in maintaining chromosome integrity and stability, and chromosome instability is associated with cancer. Through integrative analysis, this study investigates the potential value of ANKRD53 in stomach adenocarcinoma (STAD).

**Methods:**

RNA-seq and scRNA-seq data were used for integrative analysis based on online databases. Expression of ANKRD53 was confirmed by RT-PCR after bioinformatic analysis. Kaplan–Meier and Cox regression analyses were performed to evaluate the prognostic value of ANKRD53 in STAD. Gene set enrichment analysis (GSEA) was performed to evaluate ANKRD53-related signaling pathways. In addition, the interaction of ANKRD53 with immunity was also investigated.

**Results:**

RT-PCR in STAD cell lines confirmed that ANKRD53 was downregulated in STAD samples compared to normal samples in the online databases. As an independent predictive biomarker, ANKRD53 was combined with other clinicopathological parameters to create a prognostic nomogram. Using GSEA, ANKRD53 was found to be involved in five pathways, including the TGF-β signaling pathway. Further investigation revealed that ANKRD53 was associated with immune checkpoint molecules, immunological pathways, and immunotherapy, in addition to MSI, TMB and neoantigens. In addition, scRNA-seq data revealed that ANKRD53 is mainly expressed in CD8^+^ T and dendritic cells.

**Conclusions:**

ANKRD53 is an important biomarker for STAD that deserves further attention.

**Supplementary Information:**

The online version contains supplementary material available at 10.1007/s12672-024-01054-5.

## Introduction

Among the most common carcinomas, gastric cancer ranks fifth in incidence and fourth in mortality worldwide [1]. Complex interactions between microbial infections, genetics, and epigenetic factors contribute to gastric carcinogenesis [2–4]. For example, Helicobacter pylori (HP) infection is considered the leading cause of gastric cancer. However, a small percentage of infected hosts do not develop cancer, likely due to differences in other influencing factors, such as host genetics and environmental factors. 90% of gastric cancers are stomach adenocarcinoma (STAD), which are highly aggressive [3, 4]. The most common curative treatment for STAD is surgical resection. In addition, chemotherapy, radiotherapy, and immunotherapy have been shown to be effective in the treatment of STAD [5, 6]. However, the survival status of STAD is unsatisfactory [7, 8]. Therefore, the search for new biomarkers that can predict prognosis and guide individualized therapy will ultimately improve survival.

Ankyrin repeat (ANK) domains are among the most highly conserved motifs [9]. These protein domains were first discovered in the Swi6 and Cdc10 sequences of yeast cell cycle regulators [10]. ANK repeats can serve as scaffolds for protein–protein interactions [11, 12]. ANK repeat-containing proteins are involved in a variety of biological processes, including signal transduction and cytoskeletal integrity [12]. Recent research shows that the ANKRD protein family is associated with the development of many malignancies, including ANKRD13a in ovarian cancer and ANKRD22 in glioma [13, 14].

Ankyrin repeat domain 53 (ANKRD53) is a 530-amino acid protein containing three ankyrin repeats. ANKRD53 can participate in the process of chromosome segregation in mitosis and maintain chromosome integrity and stability. Seul Kim et al. [15] reported that ANKRD53 and DDA3 (differentially display activated by p53) interact during mitosis to maintain chromosome integrity. A hallmark of malignancies is chromosomal instability (CIN), and the genomes of tumor cells have been shown to exhibit various forms of genomic instability [16]. CIN refers to the increased rate of chromosome structure and number alterations found in most sporadic metastatic human tumors [17]. Bakhoum SF et al. [18] reported that CIN could promote tumor metastasis in the cover article of Nature in 2018. Thus, the role of ANKRD53 in cancer development through the regulation of CIN was significant for its mechanism. In this study, the relationships between ANKRD53 and prognosis, signaling pathways, and immunity were evaluated, especially the interaction between ANKRD53 and TGF-β signaling pathway was highlighted and discussed.

## Materials and methods

### Data collection and process

All RNA-seq data were obtained from TCGA (https://portal.gdc.cancer.gov/) (375 tumor and 32 normal tissues) for STAD. ScRNA-seq data (GSE134520 and GSE167297 datasets) of ANKRD53 were analyzed using the online TISCH2 website (http://tisch.comp-genomics.org/). Differentially expressed genes and survival curves were calculated using the R package “Limma”. The R statistical environment (R 4.4.1 software) was used to analyze all the data.

### Real-time quantitative PCR (RT-PCR) and immunohistochemistry (IHC) analysis

Cell bank of Chinese Academy of Sciences (Shanghai, China) provided GES-1, AGS and BGC-823 cell lines. After reverse transcription, RT-PCR was performed to confirm the expression of ANKRD53 mRNA. The primers for ANKRD53 were 5ʹ-AACCAGAGCCTCAGGGAAATC-3ʹ (forward) and 5ʹ-CAGGTCCACGGGAAACTTG-3ʹ (reverse). Primers for GAPDH were 5ʹ-GGAAATCCCATCACCATCTTC-3ʹ (forward) and 5ʹ-TGGACTCCACGACGTACTCAG-3ʹ (reverse). IHC was performed to confirm the expression of ANKRD53 protein. Tissue samples were sectioned. Antigen retrieval and serum sealing were performed, followed by incubation with antibody (1:500, 24283-1-AP, Proteintech). The informed consent was obtained from all the subjects, and the ethical approval (No. 83240195) for this study was granted by the Ethics Committee of Anhui Medical University. All procedures followed in studies involving human subjects adhered to national and institutional research committee ethical standards and the Declaration of Helsinki.

### Identification of independent risk factors and construction of a nomogram for prognosis

Receiver operating characteristic (ROC) curves and area under the curve (AUC) were generated using the R package survival ROC. Independent prognostic factors were evaluated by Cox regression analysis. A nomogram including ANKRD53 and other clinicopathological parameters was created using the R package "rms" to predict the prognosis in STAD. The concordance index (C-index) and AUC were used to determine the effectiveness of the nomogram. Calibration curves were also generated to graphically explore the predicted probabilities of the nomogram versus the observed events. The 45° line was found to have the best predictive values.

### Genes set enrichment analysis (GSEA)

GSEA was a critical technique to evaluate the enriched gene sets. The differences between higher and lower ANKRD53 expression cohorts were analyzed. In each test, 1000 gene set permutations were performed to determine the ANKRD53-associated signaling pathways. Significant results were defined as an FDR < 25% and an adjusted p-value < 0.05.

### Protein–protein interaction (PPI) analysis and the correlation between ANKRD53 and microsatellite instability (MSI), tumor mutational burden (TMB), and neoantigens

Genes with possible functional interactions with ANKRD53 were searched using the STRING database (https://cn.string-db.org/). The PPI network was derived based on the default conditions of the online website. The association between ANKRD53 and MSI, TMB, and neoantigens was examined using Spearman's correlation analysis, according to the Sangerbox online website (http://www.sangerbox.com/tool). The R package "fmsb" was used to create a radar map showing all relevant information.

### Immune properties of ANKRD53 in STAD

The correlation between ANKRD53 and immune cell infiltrations was evaluated using the CIBERSORT website (http://cibersort.stanford.edu/). The ESTIMATE algorithm was used to evaluate the relationships between ANKRD53 and the tumor microenvironment (ImmuneScore, ESTIMATEScore, and StromalScore). Furthermore, the co-expression analysis of ANKRD53 expression with immune checkpoint molecules or immune cell pathways was shown using the R packages "Reshape2" and "RColorBrewer".

### Evaluation of scRNA-seq data and the relationship between ANKRD53 and immunotherapy

The scRNA-seq data from the TISCH2 website (http://tisch.comp-genomics.org/) were used to investigate ANKRD53 expression at the single-cell level. According to the Tumor Immune Dysfunction and Exclusion (TIDE, http://tide.dfci.harvard.edu/), the potential effect of immunotherapy in different ANKRD53 expression groups was analyzed.

### Statistical analysis

Statistical data were analyzed using SPSS 24.0 (IBM, Chicago, USA), R4.4.1 (https://www.r-project.org/), and GraphPad Prism 6.0 (San Diego, CA, USA). The association between ANKRD53 and MSI, TMB, and neoantigens was examined using Spearman’s correlation analysis. Kaplan–Meier analysis and Cox regression analyses were used to estimate the predictive power for survival. The nomogram was generated using the R package “rms”. The "survival ROC" in the R package was used to calculate the prognostic ability using ROC analysis. A p-value of less than 0.05 was considered statistically significant.

## Results

### ANKRD53 expression and its association with prognosis in STAD

**Fig. 1:**
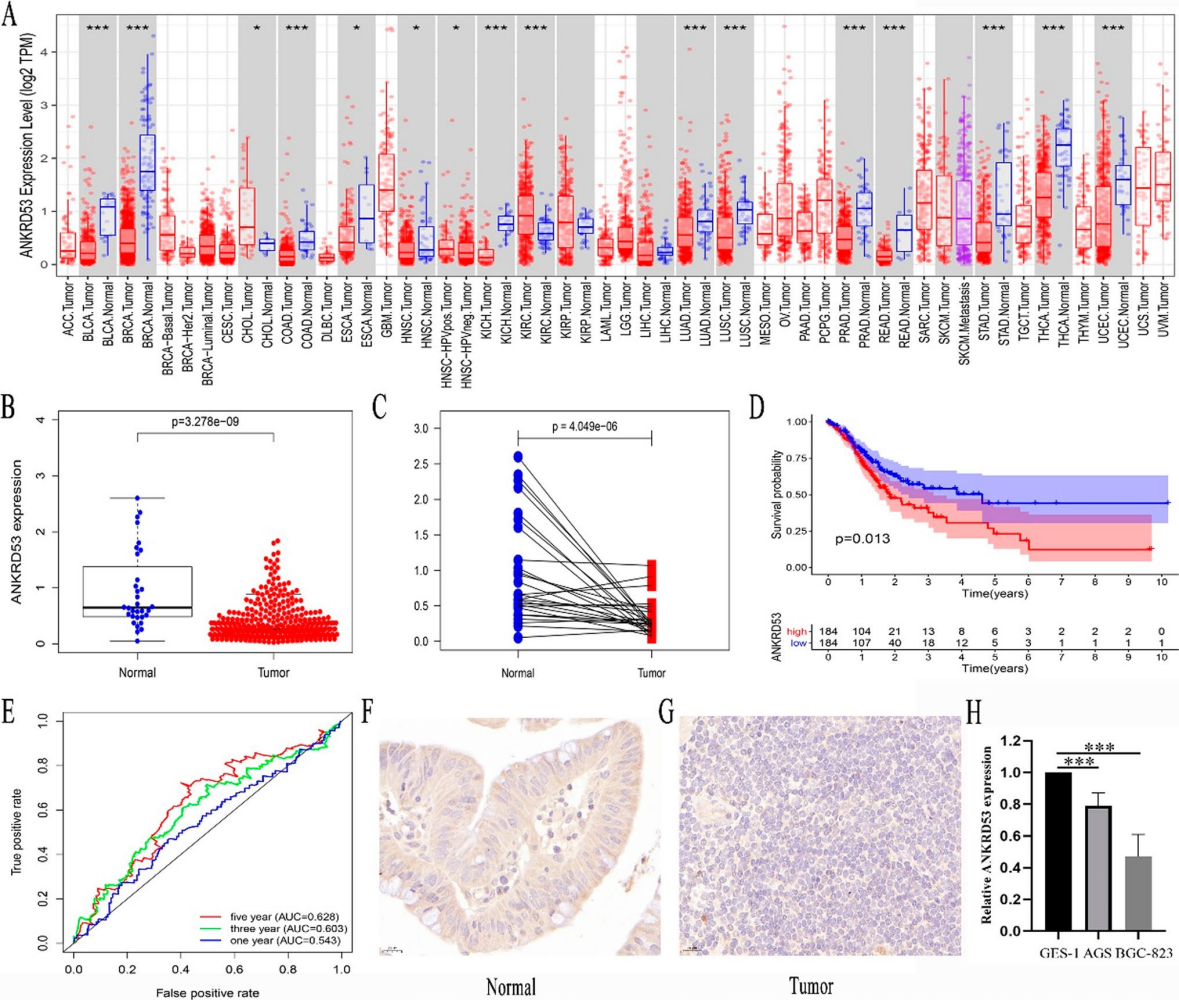
ANKRD53 expression and relationship with prognosis of patients with STAD. **A** Pan-cancer analysis. **B** Relative expression levels of ANKRD53 in tumor tissues (n = 375) and normal tissues (n = 32). **C** Pairwise boxplot of the ANKRD53 expression between tumor tissues (n = 27) and matched normal tissues (n = 27). **D** Kaplan–Meier curves for Overall survival. **E** ROC curves. IHC for ANKRD53 in (**F**) normal tissues and (**G**) tumor tissues. **H** RT-PCR analysis in STAD and normal cell lines. ***p < 0.001

We first examined ANKRD53 mRNA expression in the TCGA dataset by pan-cancer analysis. As shown in Fig. 1A, ANKRD53 expression was lower in several tumors, including STAD, compared with normal tissues. The results in STAD tissues (n = 375) and normal tissues (n = 32) were consistent with the above findings (Fig. 1B). 27 pairs of STAD tissue and normal tissue from the same patient also showed lower ANKRD53 expression in STAD (Fig. 1C). IHC results confirmed lower levels of ANKRD53 protein in STAD (Fig. 1F and G). Furthermore, RT-PCR results revealed the decreased mRNA expression of ANKRD53 in STAD cell lines (p = 0.000, Fig. 1H). Next, we investigated the predictive value of ANKRD53 in STAD. Survival analysis showed that a significant difference in survival was observed between patients with higher and lower expression groups. (p = 0.013, Fig. 1D). According to a time-dependent ROC analysis, the AUC were 0.543 (1-year), 0.603 (3-year), and 0.628 (5-year), indicating a moderate prognostic effect (Fig. 1E).

### Correlations between ANKRD53 and clinicopathological parameters

**Fig. 2:**
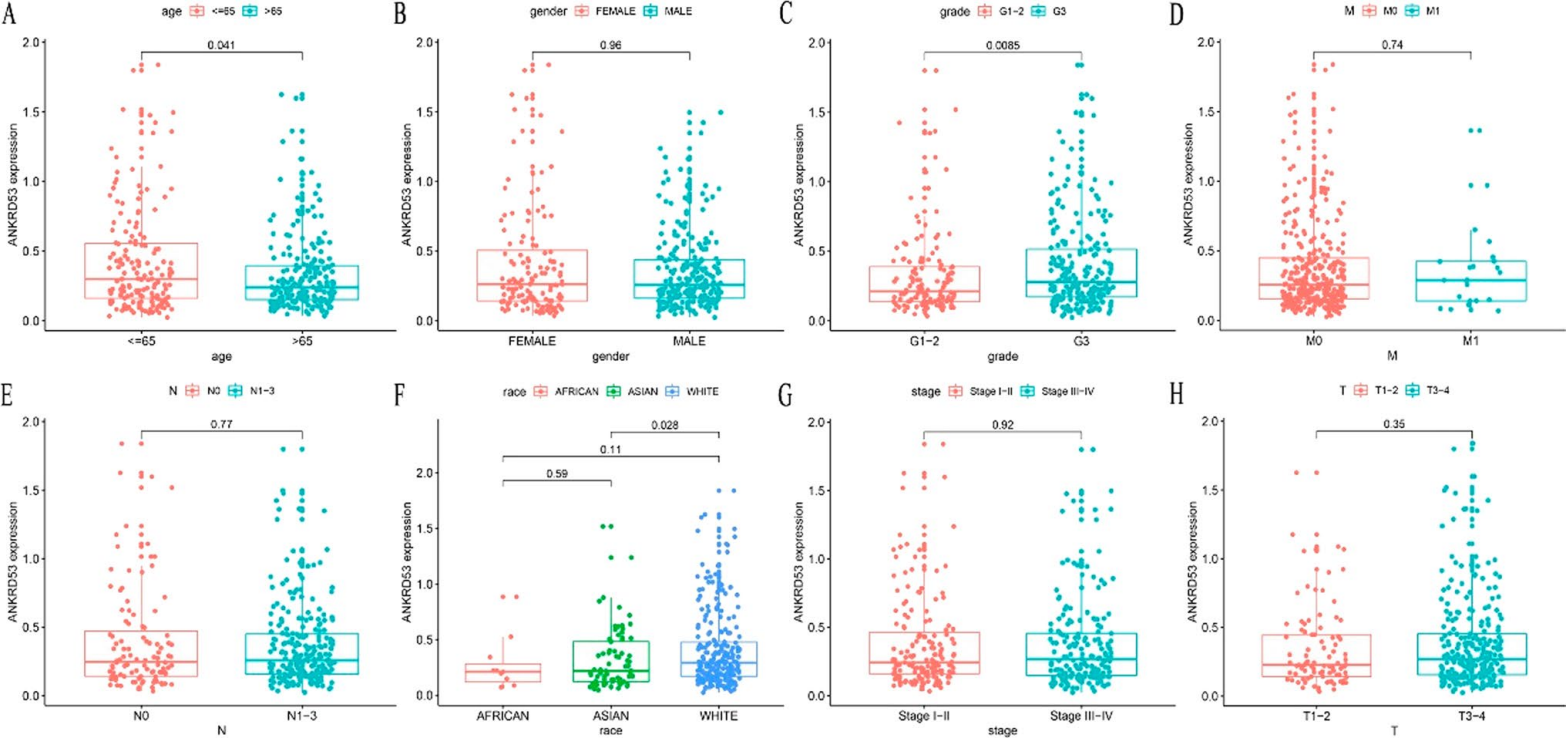
Relationships between ANKRD53 and clinicopathologic parameters. **A** Age. **B** Gender. **C** Grade. **D** M stage. **E** N stage. **F** Race. **G** Stage. **H** T stage

A t-test was performed to examine the correlations between ANKRD53 and clinicopathological parameters. According to Fig. 2, ANKRD53 expression was correlated with age (p = 0.041) and grade (p = 0.0085), while there was no correlation between ANKRD53 and gender (p = 0.96), M stage (p = 0.74), N stage (p = 0.77), T stage (p = 0.35), and pathologic stage (p = 0.92). Regarding race (Fig. 2F), ANKRD53 expression varied significantly between Asian and White (p = 0.028), but not between Asian and African (p = 0.59) or African and White (p = 0.11).

### Identification of independent risk factors and construction of a nomogram for prognosis

**Fig. 3:**
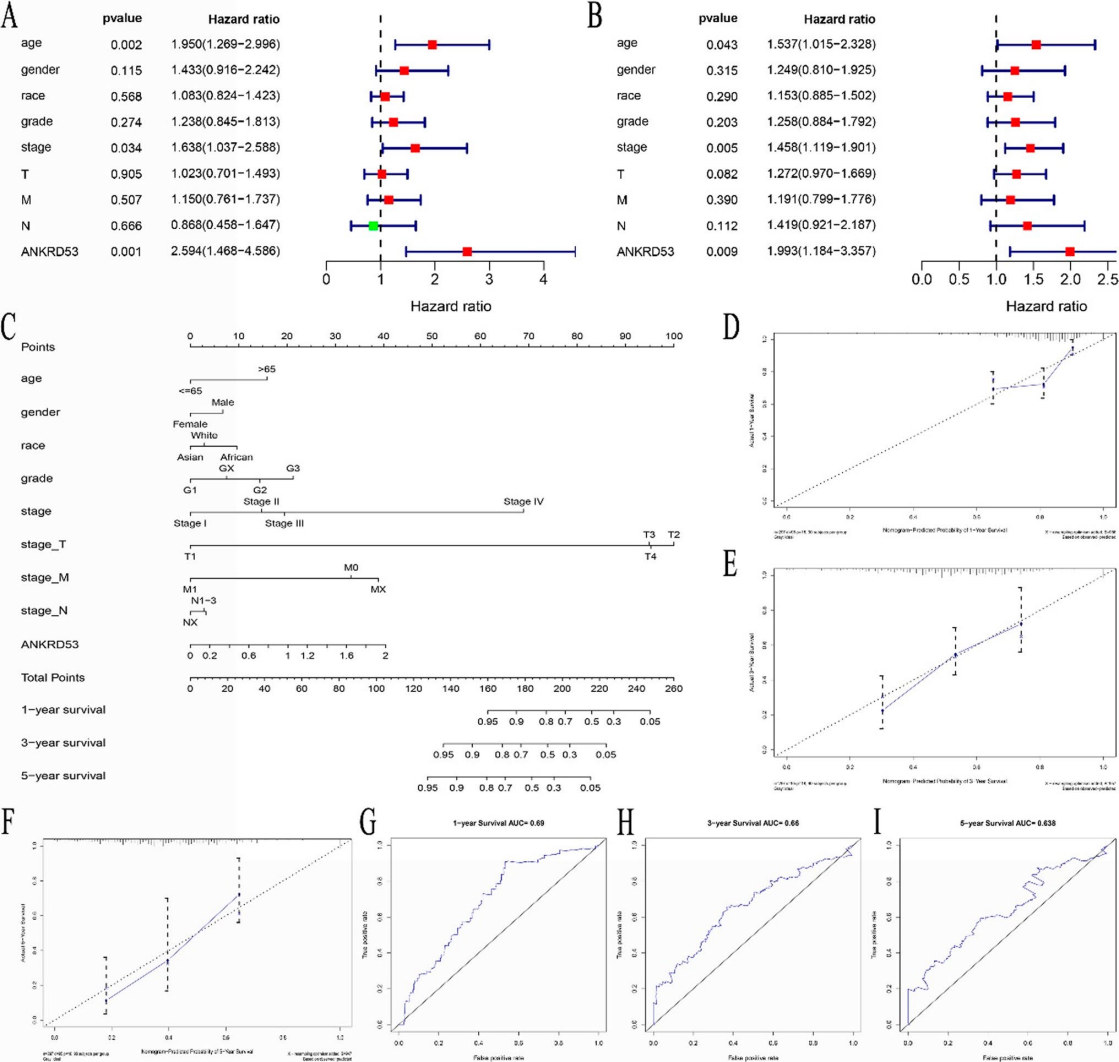
Identification of ANKRD53 as an independent prognostic factor and the nomogram construction. **A** Univariate Cox regression analysis. **B** Multivariate Cox regression analysis. **C** Nomogram for predicting prognosis of STAD. **D**–**F** Calibration curves for 1, 3, and 5 years. **G**–**I** AUC of curves of 1-year, 3-year, and 5-year survival

The prognostic value of ANKRD53 and other clinicopathological parameters was evaluated. As shown in Table S1 and Fig. 3A, univariate Cox regression revealed that age (HR = 1.537, CI 1.015–2.328, p = 0.043), stage (HR = 1.458, CI 1.119–1.901, p = 0.005), and ANKRD53 (HR = 1.993, CI 1.184–3.357, p = 0.009) were significantly associated with overall survival in STAD. Coincidentally, multivariate Cox regression showed that all the above three factors were independent risk factors for STAD (p < 0.05, Table S1, Fig. 3B). To quantitatively predict prognosis, all three independent factors were combined into a nomogram model (Fig. 3C). The C-index of the nomogram was 0.689 (Table S2). The calibration curves agreed with the prediction and observation (Fig. 3D-F). Additionally, the results of Fig. 3G-I indicated that the AUC were 0.69 (1-year), 0.66 (3-year), and 0.638 (5-year).

### GSEA analysis of ANKRD53 in STAD

**Fig. 4:**
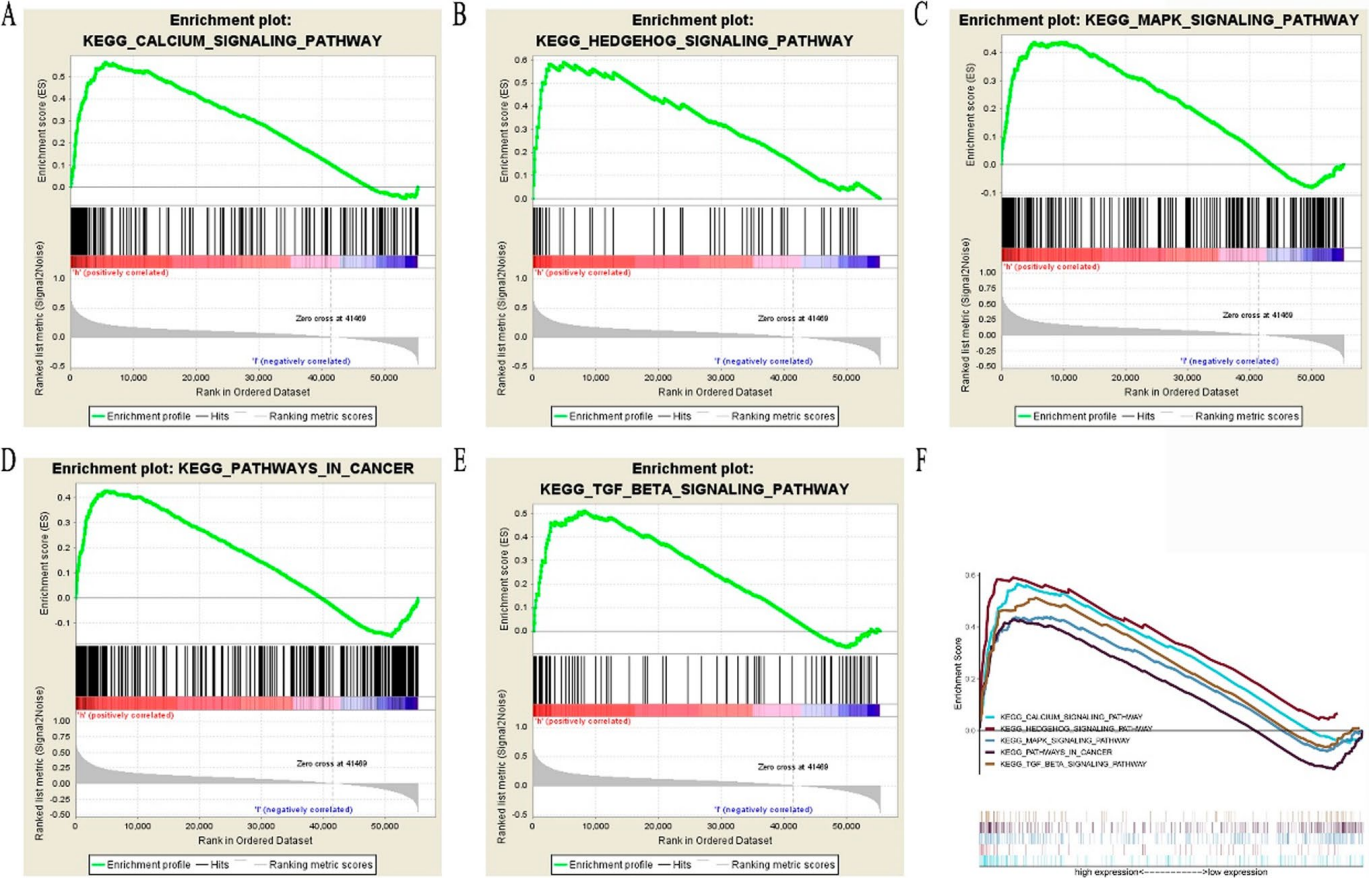
GSEA analysis of ANKRD53 in STAD. **A** Calcium pathway. **B** Hedgehog pathway. **C** MAPK pathway. **D** Pathways in cancer. **E** TGF-β signaling pathway. **F** Summary of the five significantly enriched signaling pathways

According to the GSEA analysis in Table S3 and Fig. 4, the significantly enriched signaling pathways were Calcium pathway, Hedgehog pathway, MAPK pathway, pathways in cancer, and TGF-β pathway. These ANKRD53-associated signaling pathways may affect the regulation of ANKRD53 in STAD.

### PPI analysis and the correlation between ANKRD53 and MSI, TMB and neoantigens

**Fig. 5:**
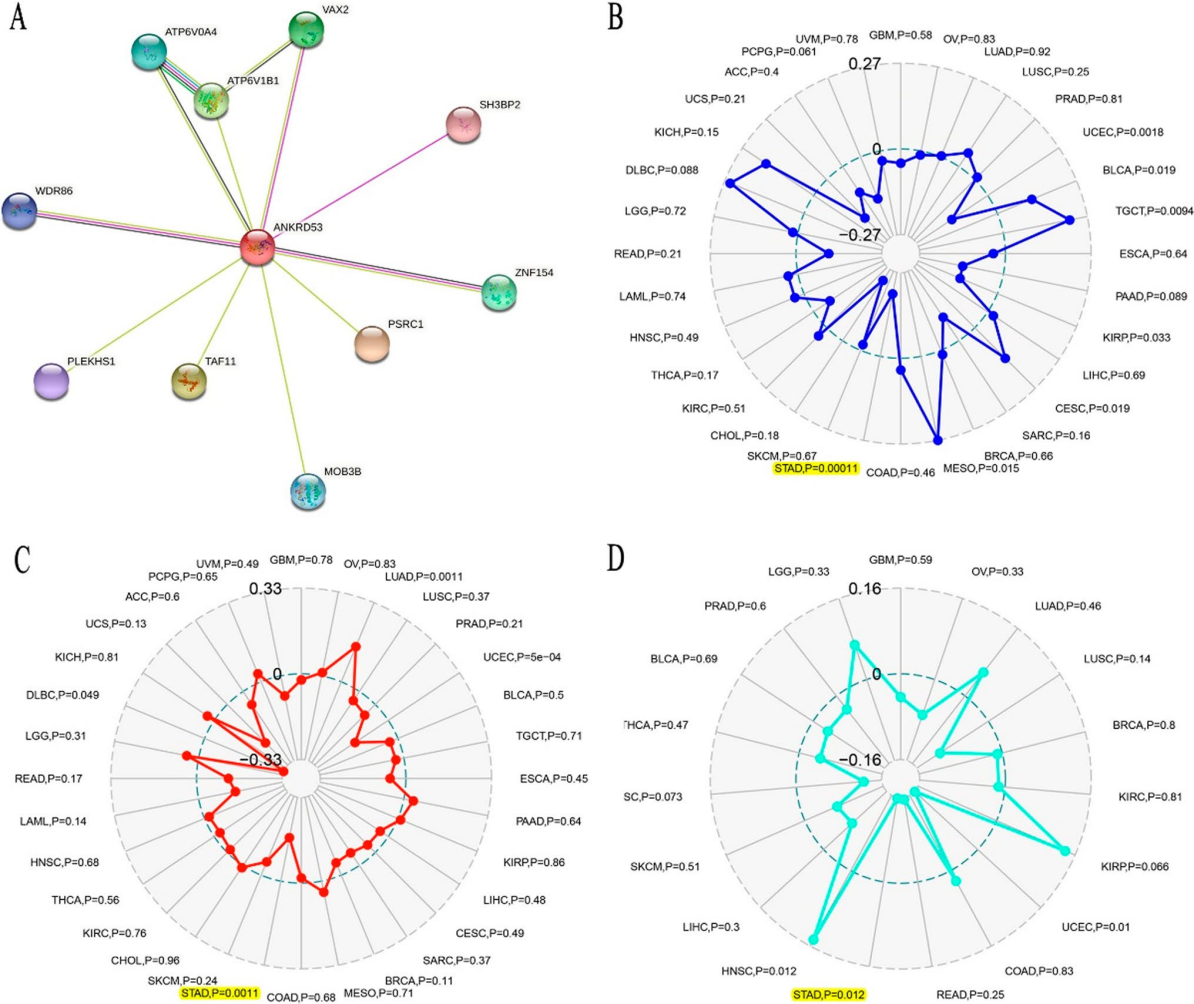
ANKRD53-related genes and relationships between ANKRD53 and MSI, TMB, and Neoantigens. **A** ANKRD53-related PPI network. Association between ANKRD53 and (**B**) MSI, **C** TMB, and **D** Neoantigens

The PPI network was constructed to investigate the ANKRD53-related proteins. ATP6V0A4, VAX2, ATP6V1B1, SH3BP2, WDR86, ZNF154, PSRC1, MOB3B, TAF11 and PLEKHS1 were functionally correlated with ANKRD53 as shown in Fig. 5A. In addition, we found that ANKRD53 strongly correlated with MSI (p = 0.00011, Fig. 5B), TMB (p = 0.0011, Fig. 5C), and neoantigens (p = 0.012, Fig. 5D).

### Immune properties of ANKRD53 in STAD

**Fig. 6:**
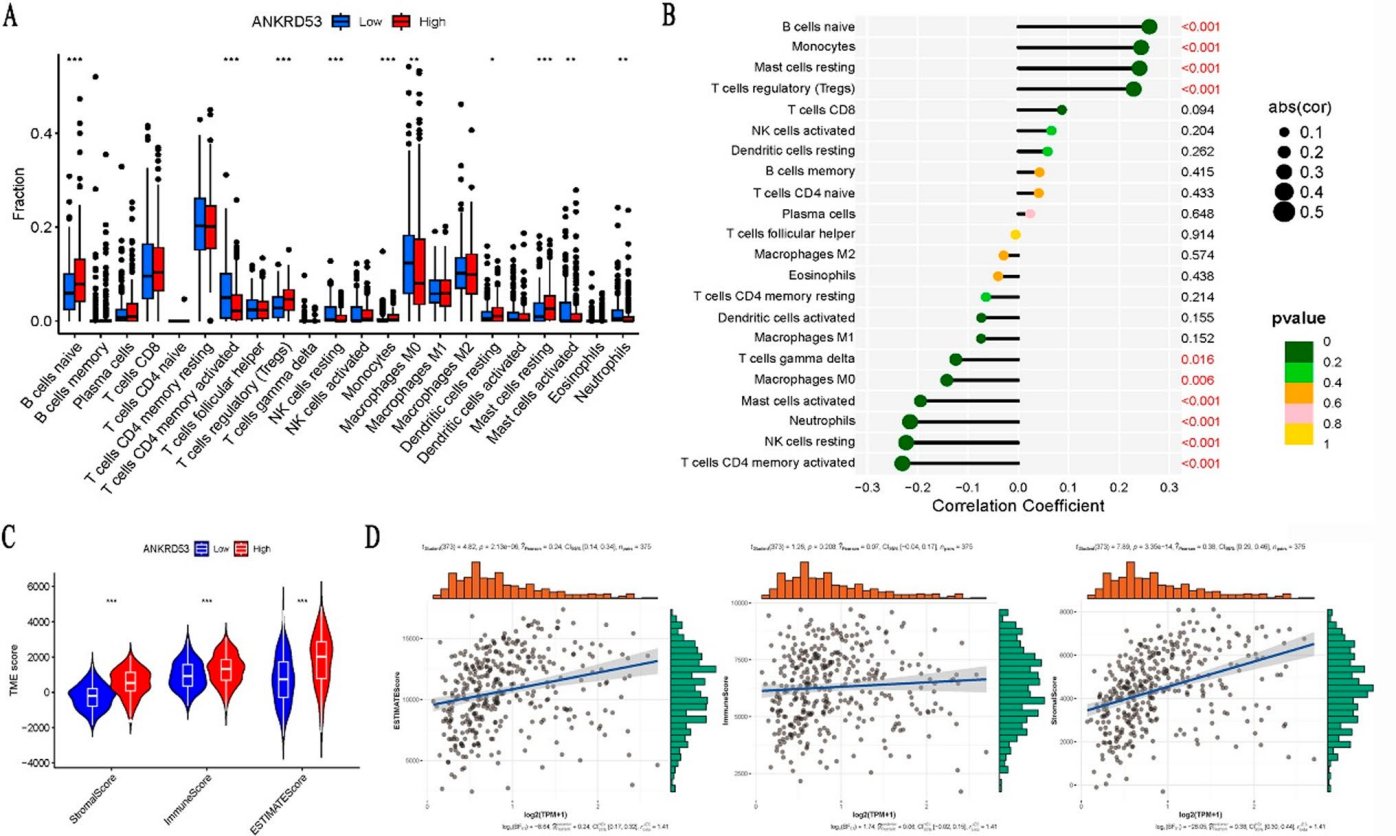
Immunological features of ANKRD53 in STAD. **A** Relationships between ANKRD53 expression and immune cell infiltration. **B** Correlation analysis of ANKRD53 and immune cell infiltration. **C** Relationships between ANKRD53 expression and tumor microenvironment. **D** Correlation analysis of ANKRD53 and tumor microenvironment

**Fig. 7:**
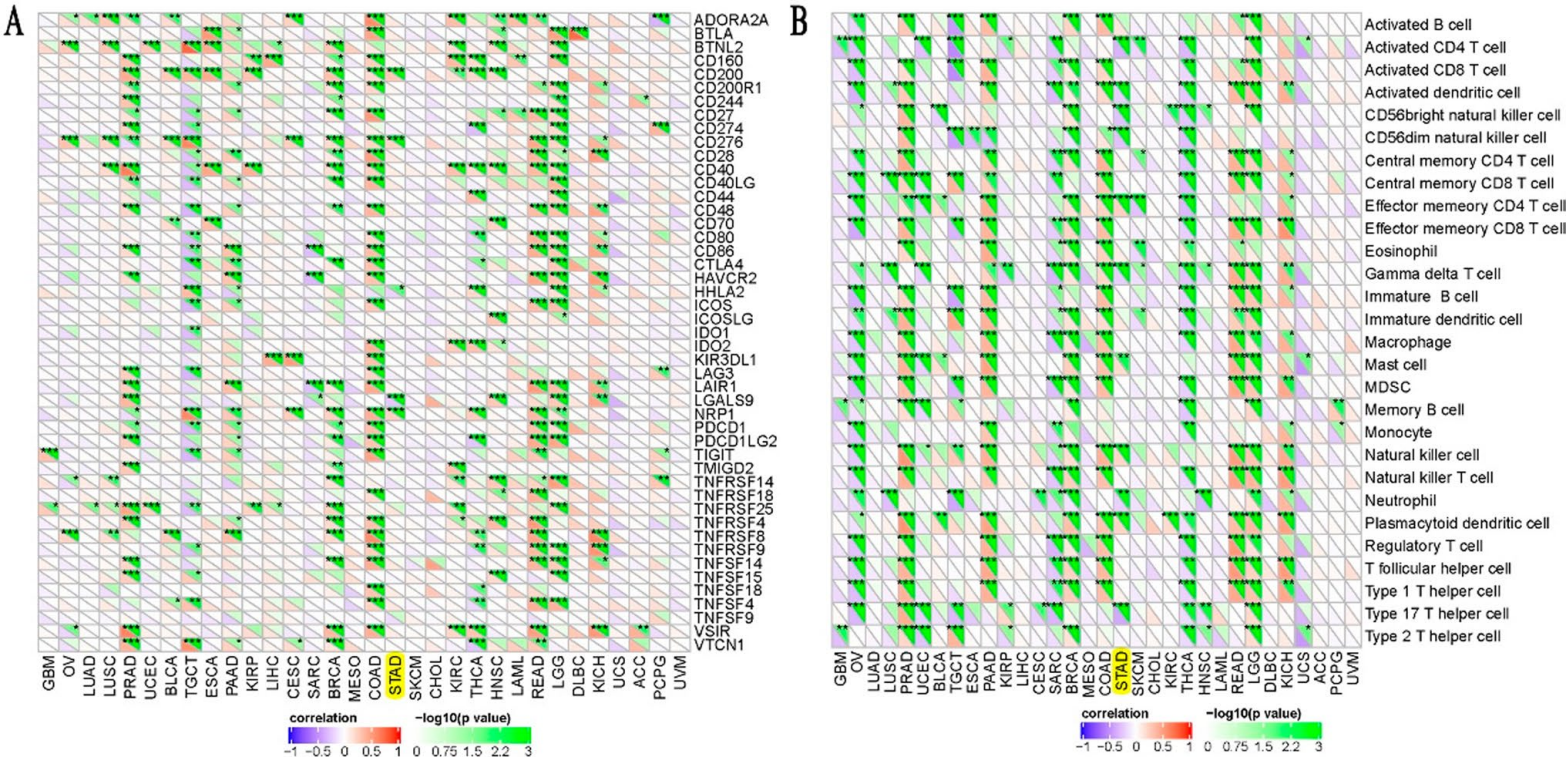
Immunological features of ANKRD53 in STAD. **A** Association of ANKRD53 and immune checkpoint molecules. **B** Association of ANKRD53 and immune cells pathways

The results of Fig. 6A and B showed that ANKRD53 was significantly correlated with the infiltration of CD4^+^ T cells, naive B cells, resting mast cells, NK cells, neutrophils, and Treg cells (p < 0.001). Regarding the tumor microenvironment (TME), significant differences in TME scores were observed in different ANKRD53 expressions between Stromal, Immune, and ESTIMATE scores (Fig. 6C). However, ANKRD53 expression was correlated with stromal and ESTIMATE scores but not with immune score in the correlation analysis (Fig. 6D). In addition, Fig. 7A results showed ANKRD53 was significantly associated with five immune checkpoint molecules including CD200, CD276, HHLA2, LCALS9, and NRP1. We also found that ANKRD53 was significantly associated with 11 different immune cell pathways, including activated CD4^+^ T cell, natural killer cell, and activated dendritic cell pathways (Fig. 7B).

### Evaluation of scRNA-seq data and the relationship between ANKRD53 and immunotherapy

**Fig. 8:**
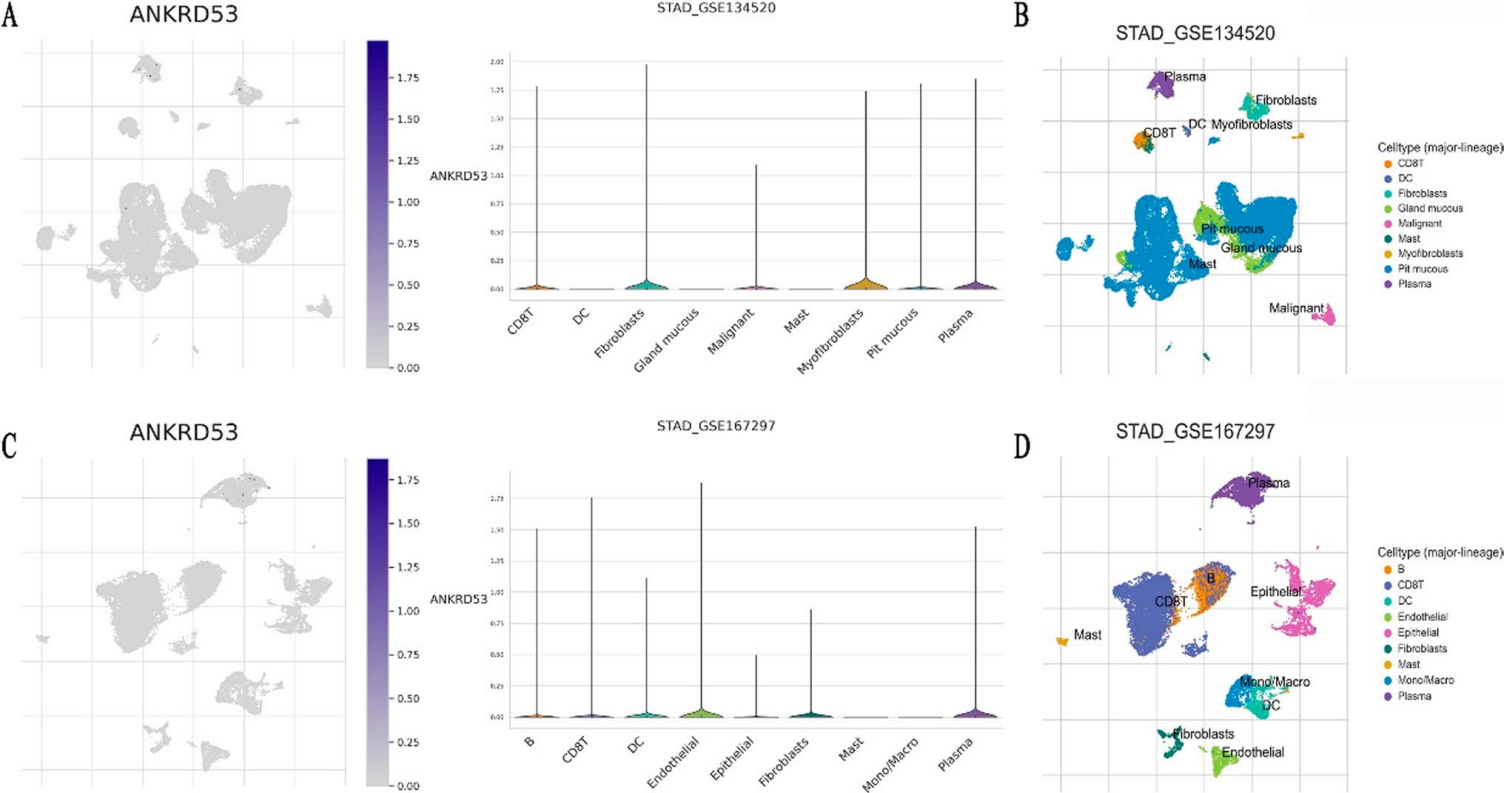
ScRNA-seq data of ANKRD53 in STAD. Umap and violin plots of ANKRD53 in STAD in (**A**, **B**) GSE134520 dataset and (**C**, **D**) GSE167297 dataset

The TISCH2 online tool was run to evaluate the ANKRD53 expression at the single-cell level. scRNA-seq data from GSE134520 (Fig. 8A and B) and GSE167297 (Fig. 8C and D) revealed that ANKRD53 was mainly expressed in CD8^+^ T cells, B cells, and dendritic cells (Figure S1A and B). To further explore the relationship between ANKRD53 and immunotherapy, immunophenoscores from the TCIA database were analyzed (Figure S2). We found that the group with a low ANKRD53 expression group had significant immunogenicity for PD1 immunotherapy (p = 0.039, Figure S2B), but not for CTLA4 immunotherapy (p = 0.11, Figure S2C).

## Discussion

Gastric cancer is a worldwide carcinoma, with an increasing incidence in Asian countries [19]. Despite significant advances in current therapies, patient survival remains unsatisfactory [7]. It is imperative to elucidate the mechanism behind the malignant growth of gastric cancer and to explore novel indicators for its early diagnosis and therapy.

As a hallmark of malignant tumors, chromosomal instability (CIN) allows tumor cells to deviate from an evolutionary path and develop resistance to a number of cancer treatments, including promoting the formation of clones that are resistant to many drugs [20]. Moreover, two articles published in Nature in 2023 showed that CIN induces epigenetic dysregulation in tumor cells, leading to epigenetic reprogramming and promoting malignant tumor progression [21, 22]. The important role of CIN in gastric cancer has been recently reported [23, 24]. Zhang et al. [25] found that gastric cancer patients with CIN were prone to HER2-positive and mucinous adenocarcinoma. Kohlruss et al. [26] investigated the use of CIN in neoadjuvant chemotherapy for gastric cancer. In conclusion, CIN offers a new direction for targeted therapy of malignant tumors. Currently, it has been reported that ANKRD53 is involved in maintaining chromosome integrity during mitosis [15]. However, there are few studies on ANKRD53. Therefore, this study analyzed the potential role of ANKRD53 in STAD for the first time to provide new ideas and theoretical basis for gastric cancer.

In this study, we first identified that ANKRD53 functions as a tumor suppressor in STAD. Its downregulation was observed not only by mRNA detection from the TCGA dataset, but also by protein detection from IHC. In addition, we used RT-PCR to confirm ANKRD53 mRNA expression in STAD cell lines. The Kaplan–Meier curves and multivariate Cox regression indicated that ANKRD53 was an independent risk factor for prognosis. We then constructed a nomogram including ANKRD53 and other independent prognostic indicators to intuitively predict the prognosis. However, the C-index of the nomogram was not satisfactory.

GSEA is a valuable method to evaluate the mechanisms of cancer biomarkers [27]. It was performed to find the potential signaling pathways in which ANKRD53 may be involved. We found that ANKRD53 was associated with Calcium, Hedgehog, MAPK, TGF-β signaling pathways, and pathways in cancer. All these signaling pathways have been reported to play critical roles in gastric cancer [28, 29]. Although many reports have shown that CIN has an important value in gastric cancer, its related mechanisms are still unclear [30]. Persistent errors in chromosome segregation during mitosis are a major cause of CIN. Recently, the TGF-β signaling pathway was found to be closely associated with cellular mitosis. According to Song et al. [31], the expression of TGF-β receptor exhibited a strong correlation with both the mitotic spindle and the G2/M checkpoint. Additionally, the formation of a complex between TGF-β receptor and the ubiquitin ligase TRAF6 was discovered to play a role in the advancement of mitosis and cytokinesis in prostate cancer cells. Moustakas et al. [32] found that the mitotic checkpoint kinase BUB1 can bind to the TGF-β receptor and modulate downstream signaling to affect mitosis. Comaills et al. [33] also found that although TGF-β-induced defects in cellular mitosis were reversible, acquired genomic abnormalities persisted, leading to an enhanced tumorigenic phenotype, and promoting malignant transformation of cells. Taken together, these findings reveal a novel function of TGF-β that is distinct from the classical signaling pathway. This opens a new research direction for cancer pathogenesis and therapy. Therefore, we speculate that ANKRD53 and TGF-β pathway may interact to regulate gastric cancer cell mitosis, which deserves further investigation in a follow-up study.

A PPI network was then constructed to explore ANKRD53-related proteins. Ten proteins were found to be related to ANKRD53.These included ATP6V0A4, VAX2, ATP6V1B1, SH3BP2, WDR86, ZNF154, PSRC1, MOB3B, TAF11, and PLEKHS1. Among these genes, silencing of SH3BP2 could affect the growth of gastrointestinal stromal tumors [34]. High expression of VAX2 was found to accelerate the development of gastric cancer [35]. PLEKHS1 has been reported as a biomarker for the diagnosis and prognosis of gastric cancer [36]. In addition, ZNF154 was found to be a tumor suppressor gene, and its low expression was associated with the poor prognosis of gastric cancer [37]. All these results indicated the potential value of ANKRD53 in gastric cancer.

MSI, TMB and neoantigens are molecular biomarkers used to evaluate the efficacy of immunotherapy and play an important role in cancer tumorigenesis [38]. We found that ANKRD53 was significantly correlated with MSI, TMB and neoantigens. Highly mutated tumors can produce a significant number of novel antigens and promote T cell infiltration. We first examined the relationship between ANKRD53 and immune cell infiltration as well as the tumor microenvironment to investigate the immunological properties of ANKRD53. The results showed that ANKRD53 was associated with the infiltration of CD4^+^ T cells, naive B cells, neutrophils, and monocytes. More and more researchers have revealed the role of innate and adaptive immune cells in immunity and immunotherapy [39–41]. The microenvironment surrounding a tumor is diverse and dynamic, consisting of multiple cell types such as stromal cells, infiltrate immune cells, and cancer cells. [42]. Our results also revealed that there were significant differences between ANKRD53 and Stromal, Immune and ESTIMATE scores.

Subsequent analysis identified that ANKRD53-related immune checkpoint molecules, including CD200, CD276, HHLA2, LCALS9, and NRP1. Deregulation of CD200/CD200R was found to contribute to gastric carcinogenesis and could be used as a novel target for immunotherapy [43]. Regarding immune cell pathways, ANKRD53 was significantly associated with activated CD4^+^ T cell, activated dendritic cell, and natural killer cell pathways. All these immune cell pathways play important roles in gastric cancer. For example, activated CD4^+^ T cells in gastric cancer were associated with the worse overall and progression-free survival in gastric cancer [44]. These observations point to the immune properties of ANKRD53.

Finally, we used the TISCH2 website to explore detailed cell type annotations at the single cell level [45]. CD8^+^ T cells, B cells, and dendritic cells exhibited the primary expression of ANKRD53. STAD is a heterogeneous malignancy with variable response to immunotherapy [46]. Lack of response to current immunotherapy is thought to be significantly associated with low tumor mutational load or low T-cell infiltration [47]. Compared to STAD patients with high ANKRD53 expression, we found that PD-1 blockade was more likely to be beneficial in individuals with low ANKRD53 expression. We conclude that there may be high levels of neoantigen and PD-L1-positive T cell infiltration in the low ANKRD53 expression group. Taken together with the above studies and results, the role of ANKRD53 in immunity may be helpful in clinical treatment decisions. Although integrative analysis was used to examine the involvement of ANKRD53 in STAD, there were still some limitations. The sample size of normal gastric tissue in the TCGA dataset was comparatively limited, which may have contributed to some bias. To fully understand the possible processes of ANKRD53 in STAD, more basic research is needed. Especially how the TGF-β signaling pathway and ANKRD53 interact to regulate mitosis in gastric cancer.

## Conclusion

In conclusion, we confirmed the downregulation of ANKRD53 expression in STAD, which was associated with prognosis. The nomogram including ANKRD53 and other clinicopathological parameters showed moderate predictive ability. Together with the immunological properties of ANKRD53, these findings suggest that ANKRD53 is a potential therapeutic target for STAD. In addition, ANKRD53 can maintain chromosome integrity during mitosis. The TGF-β signaling pathway can also regulate mitosis among the five ANKRD53-related signaling pathways. Therefore, the network mechanism of ANKRD53 and TGF-β signaling pathway in regulating mitosis and CIN in STAD deserves further experimental verification.

### Supplementary Information

Supplementary Material 1.

## Data Availability

The online data in this study are freely available from the TCGA database (https://portal.gdc.cancer.gov/). And the authors confirm that all the other data are available in the article.
