# Supplementary material for: Integrative analysis indicates the potential values of ANKRD53 in stomach adenocarcinoma
Source: Discov Oncol. 2024 May 27;15:188. doi: 10.1007/s12672-024-01054-5 (PMC11130106; doi:10.1007/s12672-024-01054-5)
Supplement: Supplementary file 1 — Supplementary Material 1. [file 12672_2024_1054_MOESM1_ESM.docx]

**Integrative analysis indicates the potential values of ANKRD53 in stomach adenocarcinoma**

**Chunjing Jin ^a,#^,** **Xu Lu ^b,#^,** **Minfeng Yang ^c,^*, Shiqiang Hou ^d,^***

^a^ Laboratory Medicine Center, The Affiliated Chuzhou Hospital of Anhui Medical University, The First People's Hospital of Chuzhou, Chuzhou, China.

^b^ Department of General Surgery, The Affiliated Chuzhou Hospital of Anhui Medical University, The First People's Hospital of Chuzhou, Chuzhou, China.

^c^ School of Public Health, Nantong University, Nantong; Department of Health Technology and Informatics, The Hong Kong Polytechnic University, Kowloon, Hong Kong SAR, China.

^d^ Department of Neurosurgery, The Affiliated Chuzhou Hospital of Anhui Medical University, The First People's Hospital of Chuzhou, Chuzhou, China.

**Corresponding author email:* [*houshiqiang@ahmu.edu.cn*](mailto:houshiqiang@ahmu.edu.cn)*; yang@connect.polyu.hk*

^#^ Chunjing Jin and Xu Lu contributed equally to this work.

**Table S1 Univariate and multivariate Cox regression analysis of ANKRD53 and clinicopathologic parameters in STAD**

| **Variables** | **Univariate analysis** | | |  | **multivariate analysis** | | |
| --- | --- | --- | --- | --- | --- | --- | --- |
|  | **HR** | **95% CI of HR** | ***p* value** |  | **HR** | **95% CI of HR** | ***p* value** |
| age | 1.537 | 1.015-2.328 | **0.043** |  | 1.950 | 1.269-2.996 | **0.002** |
| gender | 1.249 | 0.810-1.925 | 0.315 |  | 1.433 | 0.916-2.242 | 0.115 |
| race | 1.153 | 0.885-1.502 | 0.290 |  | 1.083 | 0.824-1.423 | 0.568 |
| grade | 1.258 | 0.884-1.792 | 0.203 |  | 1.238 | 0.845-1.813 | 0.274 |
| stage | 1.458 | 1.119-1.901 | **0.005** |  | 1.638 | 1.037-2.588 | **0.034** |
| T | 1.272 | 0.970-1.669 | 0.082 |  | 1.023 | 0.701-1.493 | 0.905 |
| M | 1.191 | 0.799-1.776 | 0.390 |  | 1.150 | 0.761-1.737 | 0.507 |
| N | 1.419 | 0.921-2.187 | 0.112 |  | 0.868 | 0.458-1.647 | 0.666 |
| ANKRD53 | 1.993 | 1.184-3.357 | **0.009** |  | 2.594 | 1.468-4.586 | **0.001** |

**Table S2 C-index and AUCs of the constructed nomogram**

|  | **1-year** | **3-year** | **5-year** | **C-index** |
| --- | --- | --- | --- | --- |
| **AUC** | 0.69 | 0.66 | 0.638 | 0.689 |

**Table S3 Gene set enrichment analysis results of ANKRD53 in STAD**

| **Gene Set name** | **NES** | **Nominal *p*-value** | **FDR *q-*value** |
| --- | --- | --- | --- |
| KEGG_CALCIUM_SIGNALING_PATHWAY | 2.139 | 0.000 | 0.005 |
| KEGG_HEDGEHOG_SIGNALING_PATHWAY | 1.944 | 0.004 | 0.023 |
| KEGG_MAPK_SIGNALING_PATHWAY | 1.691 | 0.012 | 0.092 |
| KEGG_PATHWAYS_IN_CANCER | 1.621 | 0.028 | 0.131 |
| KEGG_TGF_BETA_SIGNALING_PATHWAY | 1.735 | 0.012 | 0.079 |


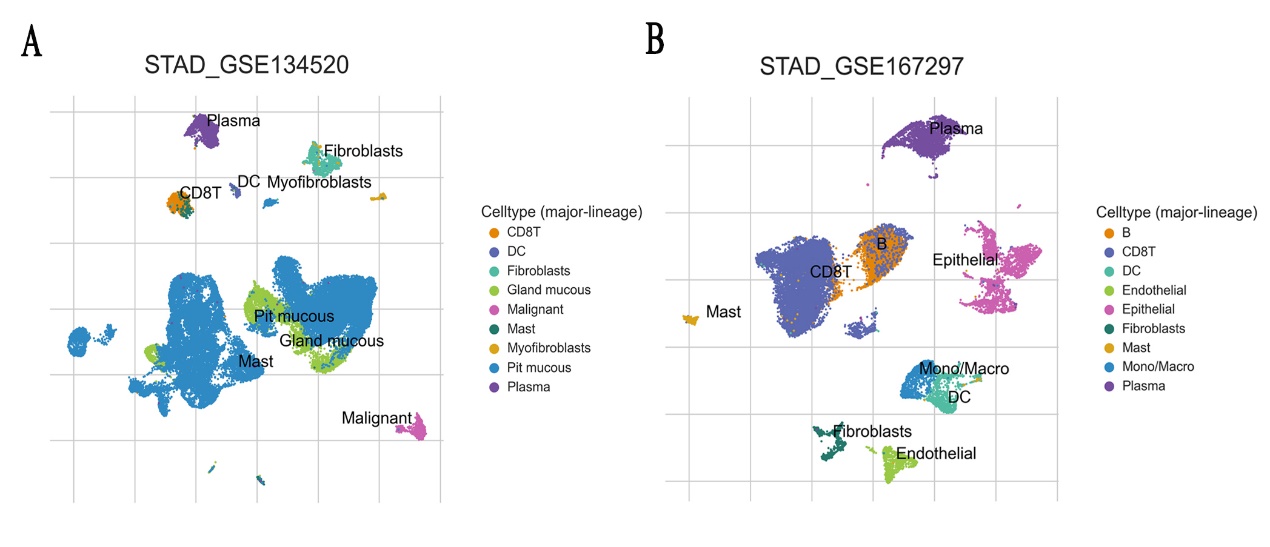


**Figure S1.** Cell clustering annotations. Cell clustering annotations of the umap plot in (A) GSE134520 dataset and (B) GSE167297 dataset.


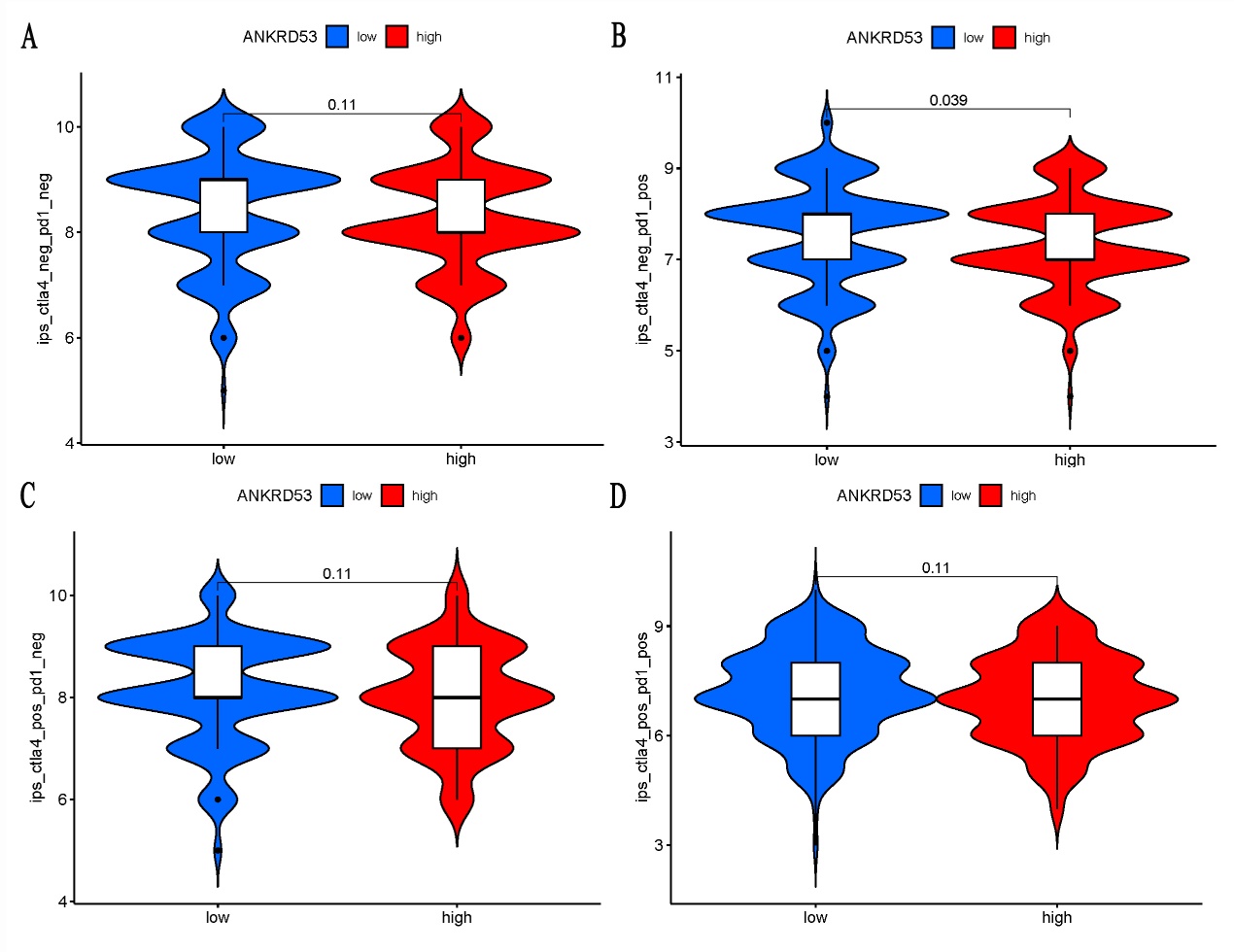


**Figure S2.** Prediction of ANKRD53-related immune responses to immunotherapy in STAD. (A-D) Distribution of ANKRD53 expression in CTLA4 scores and PD1 scores.
